# Potential and Limitations of Induced Pluripotent Stem Cells-Derived Mesenchymal Stem Cells in Musculoskeletal Disorders Treatment

**DOI:** 10.3390/biom13091342

**Published:** 2023-09-04

**Authors:** Isabelle Xavier Dias, Aline Cordeiro, João Antonio Matheus Guimarães, Karina Ribeiro Silva

**Affiliations:** 1 Teaching and Research Division, National Institute of Traumatology and Orthopaedics, Rio de Janeiro 20940-070, Brazil; linecordeiro@gmail.com (A.C.); jguimaraes@into.saude.gov.br (J.A.M.G.); 2 Laboratory of Stem Cell Research, Histology and Embryology Department, Biology Institute, State University of Rio de Janeiro, Rio de Janeiro 20550-170, Brazil

**Keywords:** cell therapy, induced pluripotent stem cells, mesenchymal stem cells, musculoskeletal disorders, orthopedics

## Abstract

The burden of musculoskeletal disorders (MSK) is increasing worldwide. It affects millions of people worldwide, decreases their quality of life, and can cause mortality. The treatment of such conditions is challenging and often requires surgery. Thus, it is necessary to discuss new strategies. The therapeutic potential of mesenchymal stem cells (MSC) in several diseases has been investigated with relative success. However, this potential is hindered by their limited stemness and expansion ability in vitro and their high donor variability. MSC derived from induced pluripotent stem cells (iPSC) have emerged as an alternative treatment for MSK diseases. These cells present distinct features, such as a juvenile phenotype, in addition to higher stemness, proliferation, and differentiation potential than those of MSC. Here, we review the opportunities, challenges, and applications of iPSC as relevant clinical therapeutic cell sources for MSK disorders. We discuss iPSC sources from which to derive iMSC and the advantages and disadvantages of iMSC over MSC as a therapeutic approach. We further summarize the main preclinical and clinical studies exploring the therapeutic potential of iMSC in MSK disorders.

## 1. Introduction

Musculoskeletal (MSK) disorders represent more than 150 diseases and conditions that affect the muscles, bones, and associated connective tissues. These conditions can cause temporary or long-term physical disability. Some disorders are characterized by chronic pain, leading to a reduction in social participation and work. Approximately 1.71 billion people live with musculoskeletal conditions, including lower back pain, neck pain, fractures, osteoarthritis, amputation, and rheumatoid arthritis. Lower back pain is the main contributor to the high prevalence of musculoskeletal conditions worldwide (570.1 million cases), followed by osteoarthritis (528 million cases), fractures (440 million cases), neck pain (222 million cases), amputation (180 million cases), gout (54 million cases), and rheumatoid arthritis (18 million cases) [1]. Disabilities caused by MSK disorders can lead to loss of productivity, work absenteeism, early retirement, and increased healthcare costs [1,2,3,4].

In recent decades, regenerative medicine for orthopedic disorders has gained attention. Non-pharmacological treatments such as cell-based therapies have generated enormous expectations regarding their capacity for tissue regeneration. Among these treatments, mesenchymal stem cells (MSC) have been the most widely studied in preclinical and clinical studies. These cells are characterized by their capacity to differentiate into three mesodermal lineages (osteogenic, chondrogenic, and adipogenic). Furthermore, MSC secrete molecules that modulate the environment, such as cytokines, growth and immunoregulatory factors, and proliferation and angiogenesis-stimulating proteins, which support tissue regeneration by regulating local immune response, inhibiting fibrosis and apoptosis and stimulating endogenous cells growth [5,6,7]. These trophic, immunomodulatory, and regenerative mechanisms are beneficial for the treatment of MSK disorders associated with osteochondral defects, cartilage lesions, and osteoarthritis [8,9].

MSC are found in postnatal tissues. The bone marrow was the first source of MSC, identified by Friedestein et al. [10]. Over the past few decades, more tissue sources of MSC have been discovered, including dental pulp and periodontal ligament [11,12], menstrual blood [13], subcutaneous adipose tissue and infrapatellar fat pads [14,15], synovial joints [16], and skeletal muscles [17]. The phenotypic characteristics of MSC from different tissues were not homogenous [18]. Its differentiation potential and proliferation rate depends on the anatomical localization [19], isolation procedures [20], and donor age [21]. Although MSC can be easily expanded in vitro, their proliferative capacity is limited because they senesce after several rounds of proliferation [22]. In addition, functional alterations such as differentiation potential, secretory phenotype, and immunomodulatory function occur during replicative senescence, limiting the number of functional cells that can be obtained in vitro for transplantation [23]. Moreover, diseased environments can negatively impact the therapeutic potential of MSCs, such as in inflammatory and age-related conditions [24,25]. Therefore, MSC have heterogeneous qualities depending on the tissue source and donor conditions, which may jeopardize MSC transplant success and hamper broader applications of MSC in clinical cell therapy trials. Alternative sources have been proposed to overcome the limited activity and availability of functional adult MSC for transplantation. In this context, induced pluripotent stem cells (iPSC) obtained from somatic cells reprogrammed with pluripotent factors are considered an alternative source of MSC. iPSC can differentiate in mesenchymal-like stem cells that share the characteristics of both iPSC and MSC. iPSC-derived mesenchymal stem cells (iMSC) possess a high proliferative capacity, with a fibroblast-like morphology, surface antigen profile, and multipotency similar to MSC [26,27]. 

Here, we review the current knowledge on iMSC as a relevant clinical therapeutic cell source for MSK disorders. We discuss why these cells have been gaining attention and, in particular, address the parameters that might affect the clinical application of iMSC. We further discuss the advantages and disadvantages of iMSC as a therapeutic tool and review examples of the main preclinical and clinical studies on the therapeutic role of iMSC in treating MSK diseases. 

## 2. iPSC Sources to Derive MSC

Pluripotent stem cells are characterized by their self-renewal and pluripotential and can be harvested from the inner cell mass of blastocysts. Since 2006, pluripotent cells have been derived from somatic cells. Pioneering studies conducted by Yamanaka et al. reprogrammed somatic cells with the pluripotency factors Oct3/4, Sox2, Klf4, and c-Myc to derive iPSC [28,29]. These iPSC overcame the ethical and immunogenic challenges associated with human embryonic stem cells. iPSC are obtained from adult somatic cells and can be autologously transplanted to prevent immune rejection. Precautions regarding the choice of an adequate cell source for inducing pluripotency are required once the cell maintains its original epigenetic status, even after reprogramming. This can be avoided via prolonged expansion in culture, although this might influence the differentiation capacity of iPSC [30,31].

Virtually all cell types in the human body have the potential to become pluripotent stem cells, albeit with considerable variability in efficiency. iPSC were first established using fibroblasts obtained from skin biopsies. More cell sources are now available such as peripheral blood cells, bone marrow and epithelial cells, stomach and liver cells, melanocytes, and neural stem cells [32,33,34]. 

iMSC derived from different cell sources demonstrated varied characteristics in differentiation potential. A study using fibroblasts from different sources to derive iMSC (gingival, periodontal ligament, and lung) demonstrated that although all lineages underwent osteogenic differentiation, presenting mineralized deposits in vitro, the iMSC derived from periodontal ligament had an increased capacity to form mineralized structures [35]. Little evidence supports the ultimate cell source from which to obtain iMSC with specific applications in MSK disorders. Therefore, the decision of which cell source to choose should be based on essential criteria for clinical application in general: it should be easy to isolate with minimally invasive procedure; easy to culture and expand to enable reprogramming in a short period of time; and abundant in the tissue [36].

The cell sources used to derive iPSC and further differentiation into MSC to evaluate its potential to treat MSK disorders are summarized in Table 1. Cell sources used to derive iPSC in the studies cited were the following: fibroblasts; CD34+ bone marrow cells; HEK239T cell line; and adipose-derived stem cells. However, a few studies did not mention the iMSC cell source.

Fibroblast is the main cell type used in iPSC studies. It can be obtained from skin punch biopsy and foreskin after circumcision procedure [54,55]. The biopsy area is prepared in a sterile fashion using 70% ethanol and 1% lidocaine with epinephrine for anesthesia [56]. As listed in Table 1, eight studies used these cells to derive iMSC. Four studies used a cell lineage (iPS-S-01) established from human newborn foreskin fibroblasts. After maintaining iPSC in culture medium for 14 days, it was replaced with a serum-free MSC culture to obtain cells with iMSC characteristics. These cells or their derivatives (exosomes and extracellular vesicles) ameliorated tendinopathy in rats [37,38,39] and attenuated intervertebral disc degeneration [40]. One study derived iPSC from human dermal fibroblasts and used the embryoid formation method to differentiate iPSC in iMSC. These cells successfully repaired bone defects in mice. [41]. Fibroblast was also used to generate iPSC cultivated in a feeder and xeno-free system which were differentiated in MSC for osteogenic differentiation and could regenerate calvarial bone defect in vivo [42]. Human fibroblasts were also used to derive iMSC that underwent two different pathways of differentiation. These were effective in an experimental model of osteoarthritis [43]. Human fetal foreskin fibroblasts were also used to derive iPSC in a study of critical sized bone defect in pig models [44].

Bone marrow CD 34+ cells obtained from healthy adult donors are used as sources of iPSC. Mononuclear cells are isolated using a standard gradient protocol, and CD34+ cells are selected through magnetic-activated cell sorting (MACS). After reprogramming, iPSC were differentiated in MSC by embryoid body formation. These iMSC underwent in vitro angiogenic and osteogenic differentiation when seeded on a calcium phosphate scaffold and promoted in vivo mineral synthesis in rat cranial defect [45,46,47,57].

HEK293T cell lineage and adipose stem cells were also used as source cells in two preclinical iMSC studies. HEK293T is an immortalized human embryonic kidney cell line. iPSC reprogrammed from HEK293T were differentiated in MSC via embryoid body formation method. iMSC transplanted to a osteonecrosis experimental model promoted bone repair and prevented bone loss [48].

Equine adipose derived stem cells were isolated from abdominal areas for iPSC generation. Afterward, iPSC were differentiated in MSC by changing the culturing medium and transplanted in horses with musculoskeletal disorders. The study demonstrated generally positive effects, such as reducing lameness. However, three animals presented severe adverse events [51].

## 3. Methods to Derive iMSC from iPSC

Several culture methods have been applied to generate MSC from iPSC [58]. The main differentiation methods to derive MSC from iPSC are as follows: MSC switch; embryoid body formation; specific differentiation; pathway inhibitor; and platelet lysate. The MSC switch method consists of replacing the iPSC culture medium with the MSC culture medium. In the embryoid body formation method, cells were cultured in clusters forming embryoid bodies and then seeded in MSC-specific culture medium. The specific differentiation protocol generates a precursor before differentiation in MSC. iPSC are differentiated in neural or cardiac progenitors (priming of iPSC); for example, before differentiation in MSC. Authors that use this protocol argue that these precursors are more similar to MSC than iPSC. The pathway inhibitor method consists of blocking signaling pathways with chemicals, such as inhibitors of TGF-beta and p38-MAPK. This inhibition leads to the downregulation of pluripotency genes and differentiation in MSC. The less popular method is the use of platelet lysate to differentiate iMSC from iPSC. 

From the above cited methods, those used in preclinical trials aiming to treat MSK disorders were as follows: MSC switch; embryoid body formation; pathway inhibitor; and specific differentiation. In the MSC switch method, iPSC were initially incubated in iPSC culture medium for several passages. Afterward, the medium was replaced with an MSC culture medium. Cells were then cultivated until passage 3 to 11 for further experiments. MSC obtained with this method were able to ameliorate tendinopathy [37,38,39], intervertebral disc degeneration [40], osteoarthritis [49], and bone fracture [50]. Another MSC switch method approach is the serial plate passage. Briefly, iPSC medium is changed to MSC induction medium for one passage on gelatin-coated plates and then transferred for an additional passage to a second gelatin-coated plate. After, the cells are transferred to an uncoated plate until passage 3–11. MSC obtained using this method were used to treat musculoskeletal disorders in horses [51]. 

In the embryoid body formation method, embryoid bodies are formed and cultured in suspension in low attachment plates. Afterward, they were transferred to a gelatin-coated plate with MSC induction medium until cells dissociate and adhere to the substrate. Cells were then transferred to flasks and subcultured for further experiments. MSCs obtained through this method were able to repair bone calvarial defects [42,45,46,47], promote bone repair in an osteonecrosis model [48], and showed potential to regenerate muscle in a Duchenne muscular dystrophy model [52].

The pathway inhibitor method consists of using an inhibitor that facilitates mesenchymal differentiation. A study using iMSC derived via this method investigated its effect on critical-sized bone defects. The iPSCs were initially cultured in Matrigel with iPSC medium. After, the medium was replaced by MSC medium supplemented with the TGF-β pathway inhibitor SB 431542 for 14 days to facilitate the transition from epithelial to mesenchymal cells. Cells were then reseeded on uncoated dishes and characterized as MSC after several passages [44].

Finally, the specific differentiation method used in an experimental model of osteoarthritis consisted of iPSC incubation in an induction medium for differentiation in mesoderm and neuroepithelial-like cells. Briefly, iPSC formed embryoid bodies that were then transferred to collagen-coated dishes containing medium for specific mesodermal or neuroepithelial differentiation. The derived MSC could regenerate cartilage in osteoarthritis [43].

Although several methods to derive iMSC exist, there is no evidence for which method would be more suitable for therapeutic application in MSK disorders. More studies comparing different methods of iMSC differentiation in orthopedic diseases are necessary. 

## 4. iMSC Therapeutic Potential—Advantages and Disadvantages

MSC from adult tissues are scarce, and their availability in sufficient numbers for therapeutics require extensive expansion in culture for subsequent applications. The prolonged culture can cause replicative senescence [59]. Furthermore, MSC are a heterogeneous population composed of clones with different predisposition for mesodermal lineages [60], which can limit the development of standard protocols for clinical application [25]. Thus, in recent years, researchers have been looking for new sources of MSC. The generation of iPSC from adult somatic cells following differentiation into MSC offers the possibility of generating a high yield of patient-specific MSC, which can be derived from a single iPSC clone, reducing heterogeneity [61].

The generation of large quantities of cells is fundamental for cell therapy-based protocols. iPSC are an inexhaustible source of MSC because they proliferate indefinitely in a pluripotent state without signs of replicative senescence, which can provide the large cell numbers required for clinical therapies. iMSC are capable of proliferating in culture for 120 population doublings through approximately 40 passages without entering replicative senescence or plasticity loss, whereas MSC generally undergo culture senescence after 8–10 passages [62]. Moreover, a recent study on donor-matched comparisons between iMSC and primary MSC (all lineages obtained from bone marrow-derived MSC) revealed that iMSC were more proliferative and exhibited longer telomere than their parental MSC. The cumulative cell number after more than 40 passages was approximately a thousand times greater in two out of three iMSC lineages than that in primary MSC [63]. Additionally, no considerable differences regarding endothelial–mesenchymal transition (EMT)-related genes and pluripotency-related genes were observed [63].

MSC present donor-dependent variations on cell proliferation, immunomodulatory functions, and differentiation potential in vitro and in vivo, even when obtained from the same tissue, explaining inconsistencies and variability in clinical outcomes [25,64,65,66]. In contrast, iMSC originating from a single iPSC clone are homogenous compared to MSC obtained from various human tissue sources. Their molecular signatures are consistent among different batches and their biological performance is stable [64]. Additionally, native MSC present aging-related gene patterns, whereas iMSC acquire a rejuvenation-associated gene signature during the reprogramming process—regardless of donor age—in which cells are reversed into a more embryonic state through epigenetic and chromatin remodeling, expressing genes similar to those expressed in pluripotent stem cells but not in adult MSC [67]. Notably, iMSC have overlapping expression of developmental biological process-related genes with native MSC of a young age, such as fetal- and umbilical cord-derived MSC [61,67]. Donor-matched comparisons of iMSC and primary MSC revealed transcriptomic changes in the transition from primary MSC to iMSC regarding biological processes that control cellular function response to stimuli or developmental processes, indicating that iMSC function as an independent entity with respect to their parental MSC [63]. In addition to development phenotype differences, iMSC showed increased expression of the pericyte markers NESTIN and CD146, indicating that cell fate changes towards pericyte-like cells during MSC reprogramming to iMSC [63]. As several genes within the rejuvenation signature are associated with early development, iMSC may have enhanced regenerative properties over adult native MSC [45,61,67]. Wang et al. compared the bone regeneration potential of human-derived iMSC, umbilical cord MSC, and native bone marrow MSCs seeded on biofunctionalized macroporous calcium phosphate cement (CPC) in a rat model of cranial defect [45]. All types of MSC showed osteogenic genes upregulation and mineral synthesis in vitro. The in vivo new bone area fractions at 12-weeks after implantation were similar among the three types of MSC [45]. Moreover, DNA methylation patterns of iMSC was similar to those of bone marrow-derived MSC, and the bone repair and preventing bone loss effectiveness of iMSC was equivalent to primary MSC in an osteonecrosis rat model [48]. Similar to primary bone marrow-derived MSC, iMSC generated via lateral plate mesoderm repaired osteochondral defects [43]. It remains to be elucidated whether the distinct development phenotype of iMSC confers a higher therapeutic potential over native MSC on in vivo MSK disease models. Moreover, deciphering the differentiation routes of iMSC will contribute to increasing the clinical efficiency of iMSC [68]. It is important to note that the exact development phenotype of iMSC may vary depending on the cell source and specific methods used to generate and differentiate them [61]. Further research is needed in order to gain a deeper understanding of the developmental phenotype of iMSC derived from adult tissues and its impact on their regenerative potential in treating MSK disorders. 

The use of allogeneic MSC is considered safe; however, clinical adverse effects after transplantation in equine models, such as increased pain and cellular infiltrate in synovial joints, edema, lameness, and flush, have been observed [51,69]. The major histocompatibility complex (MHC) mismatched MSC cells are not immune privileged, as cell-mediated, humoral, and in vivo rejections occur; nevertheless, these studies only used animal models [70]. In contrast, iMSC have the potential for patient-specific autologous therapies. Furthermore, the immunogenicity of differentiated tissue (skin and bone marrow) derived from iPSC elicits limited immune responses as T cell infiltration [71]. 

Some studies have compared the biological characteristics of MSC and iMSC related to their regenerative potential. A recent study on the donor-matched comparisons between iMSC and primary MSC revealed that iMSC consistently secrete higher amounts of growth factors, cytokines, and proteins associated to cell–cell and cell–extracellular matrix interactions than their parental MSC [63]. This is particularly important for regenerative medicine approaches based on the use of MSC, as a main mechanisms used by MSC to regenerate tissues involve the paracrine signaling of bioactive factors and signals that are secreted by MSC at variable concentrations in response to local microenvironmental conditions [7]. In addition, iMSC have similar immunomodulatory potential to MSC. The periodontal and gingival tissue-reprogrammed cells to iMSC could suppress effector T Cells and Th1/Th2 cells and stimulate Treg cells proliferation similarly to MSC [72]. Canine iMSC demonstrated similar immunomodulatory properties to MSC from bone marrow and adipose tissue. Regarding immunomodulatory and pluripotency factors, iMSC transcriptomes are more similar to MSC than to the iPSC from which they were derived [73]. A recent study on the donor-matched comparisons of iMSC and primary MSC revealed that iMSC exerted more potent immune suppression of both CD4+ and CD8+ cell proliferation on allogeneic immune stimulation [63]. Accordingly, iMSC were more potent in inhibiting NK cell proliferation and function than parental MSC [74]. It remains to be elucidated whether these distinct immune-suppressive activities of iMSC could be advantageous in cell therapeutic trials in which immune-modulating effects may control inflammatory diseases affecting the musculoskeletal system. 

In addition, iMSC demonstrated a superior capacity for inducing tissue cell proliferation. For example, one study demonstrated that iMSC-derived exosomes injected intra-articularly in a mouse model of collagenase-induced osteoarthritis stimulate higher chondrocyte proliferation than exosomes of synovial membrane-derived MSC [49]. This is advantageous in the clinical application of iMSC for cartilage regeneration. 

Pluripotent stem cells are known for their tumorigenic potential. Nonetheless, iMSC can bypass teratoma formation caused by the differentiation of iPSC, which is a major concern in regenerative medicine. iMSC reportedly lack tumorigenicity when transplanted to immunodeficient mice [75,76,77]. When differentiating iPSC in MSC, caution is needed in order to ensure that every single cell has been differentiated. Optimized protocols are required in order that all cells in the final therapeutic product have differentiated in MSC (all pluripotent cells must differentiate in MSC, otherwise the cell product can become tumorigenic) [78]. Culture methods to avoid risks of tumor formation have been developed. Bloor et al. developed a manufacturing process involving three steps: (1) after mesoderm differentiation induction, cells were cultured in a semi-solid medium that did not support iPSC survival; (2) filtration in a mesh to eliminate small clumps of undifferentiated cells; and (3) expansion in plastic-adherent cell conditions that favor iMSC growth [79]. Eto et al. selected PDGFR-α+ and VEGFR- cells using FACS after mesodermal and neuroepithelial differentiation [43]. This cell purification step was incorporated because a previous report has demonstrated that PDGFR-α–cells formed teratoma more frequently than PDGFR-α + cells [80]. For the induction of somatic cells into pluripotent stem cells, technologies other than non-viral based factors should be considered for reprogramming, i.e., chemical-, plasmids-, and recombinant protein-based approaches. Thus, ensuring the complete differentiation of every single cell is crucial iPSC differentiation to MSC; failure to do so can lead to potential tumorigenicity, emphasizing the need for protocol optimization. Additionally, exploring alternative reprogramming technologies may offer viable options for achieving safe applications. Although clinical trials have not reported MSC-derived tumor development in patients, these studies neither confirmed nor excluded the risk of tumorigenicity in patients [81]. 

The development of iPSC-based therapies is labor-intensive and involves high production costs. Production costs for the establishment of autologous master banks can be increased due to the need for individual screening of infectious agents. Allogeneic banks are more cost-effective because samples from a single donor can be batched for reliable screening tests [82]. iPSC can proliferate indefinitely, and a master cell bank from a single healthy donor is sufficient to generate MSC in large quantities for allogeneic therapy. In addition, MSC from tissue sources are limited by the availability of donors and their cell proliferation capacity [64].

## 5. Preclinical and Clinical Studies in MSK Disorders Using iMSC 

Preclinical studies have used animal models to examine the effects of iMSC in MSK disorders. Table 1 summarizes the main studies of these cells in MSK disorders. 

In a comparative study, iMSC seeded in a calcium phosphate cement scaffold in a rat cranial defect showed good cell viability, promoted de novo bone regeneration, and increased bone vessel density, similar to the effects of Umbilical Cord Mesenchymal Stem Cells (UCMSC) and Bone Marrow-derived Mesenchymal Stem Cells (BMMSC). Immunohistochemistry demonstrated that these cells contributed to bone formation in cranial defects in rats. Regarding osteogenic differentiation, iMSC showed similar mRNA levels of osteogenic markers, including alkaline phosphatase *(ALP)*, runt-related transcription factor 2 (*RUNX2*), and type 1 collagen (*COL1*), although osteonectin (*OC*) was downregulated compared with UCMSC and BMMSC [45]. 

Recently, the implantation of chondrogenically differentiated human iMSC into a 2-mm radial bone defect in nude mice promoted a high rate of bone union (100% and 70% for groups treated with iMSC derived from two different iPSC clones, respectively), which was significantly higher than the rate found in the control group (18%). Histological evaluation revealed a transition from hypertrophic cartilage to newly formed woven bone, which supports the use of iMSC-based cartilage grafts to repair large bone defects via endochondral bone ossification [53].

Concerns about possible teratogenic properties have been debated; thus, some authors prefer to use cell-free therapy using exosomes or extracellular vesicles derived from iMSC. These cell products associated with β tricalcium-phosphate (β-TCP) scaffolds promoted bone regeneration in a critical-sized bone defect in female rats with ovariectomy-induced osteoporosis. Vessel area and osteogenic markers were increased in bone defects which were correlated with exosome concentration on β-TCP scaffold [50]. iMSC exosomes were also used in an experimental model of osteoarthritis. In a scratch wound in vitro assay, iMSC exosomes had a better effect on human chondrocytes migration than exosomes obtained from synovial membrane-derived MSC. Moreover, histological analysis of the medial tibia plateau demonstrated that iMSC exosome had a positive effect on cartilage degradation repair, measured using the OARSI cartilage osteoarthritis histopathology grading system [49]. In a recent study, carrageenan-induced tendinopathy was alleviated via iMSC exosome injection in the tendon quadriceps. The morphological analysis demonstrated that treated tendons had continuous and regular arrangements compared with animals that received the vehicle. Furthermore, iMSC exosomes inhibit mast cell infiltration and are associated with nerve fibers, as evidenced by the immunofluorescence staining of the quadriceps tendon with tryptase and PGP9.5 [37]. Using the same tendinopathy animal model, iMSC-derived extracellular vesicles reduced pro-inflammatory cell infiltration (M1 macrophage) and cytokines (IL-1β, TNF-α, IL-6) in diseased tendons, thereby mitigating inflammation, inhibiting capillary proliferation, and rescuing tendon from degeneration [38]. iMSC large extracellular vesicles (lEV) can attenuate pain and inflammation in a tendinopathy model by delivering the proteins DUSP 2 and DUSP 3 and regulating the p38 MAPK signaling pathway, favoring the polarization of M1 macrophage to M2 macrophage phenotype [39]. Small extracellular vesicles (sEV) can also be obtained from iMSC. Attenuation of intervertebral disc degeneration was demonstrated in an experimental model. iMSC-sEV upregulated *Sirt 6* in the nucleus pulposus cells in vitro, alleviating age-related dysfunction and senescence by delivering miR-105-5p [40].

iMSC developed via different differentiation methods could have different therapeutic applications. A study differentiated iPSC in MSC using two methods: mesodermal differentiation (PSP-MSC); and neuroepithelial differentiation (RA-Pα-MSC). Cells were then applied to three experimental models: skin wound; ulcer pressure; and osteoarthritis. iPSP-MSC had better outcomes in the experimental model of skin wound healing than those of RA-Pα-MSC. In contrast, RA-Pα-MSC were more effective in treating pressure ulcers. Although there is a difference between the two experimental models, both methods could improve cartilage degeneration in the osteoarthritis model. Cartilage degeneration was measured through a histological analysis (Mankin score) which evaluated the alignment of chondrocytes and the staining of intracellular and paracellular regions [43]. 

Cocultivation of iMSC with endothelial cells increases their therapeutic potential. iMSC co-cultured with HUVEC were used to vascularize calcium phosphate cement (CPC) scaffolds before implantation into bone defects in vivo. The cultured scaffold demonstrated microcapillary-like structures, enhanced mineralization, and showed an increased percentage of new bone area formation after transplantation in rats [46]. A similar study demonstrated that iMSC co-cultivated with HUVEC in CPC scaffolds had a similar bone regenerative capacity as BMMSC, UCMSC, and embryonic stem cell-derived MSC (ESC-MSCs) [47].

A robust study has identified two subpopulations of iMSC. In the embryoid bodies formation differentiation method, the cells that attached earlier were named early iMSC (aiMSCs), and the cells that attached later were named late MSC (tiMSCs). These two subtypes were transfected with BMP-6, and the capacity for orthotopic and ectopic bone formation was analyzed. Both cell subtypes were less efficient in inducing ectopic bone formation in vivo than BMMSC. Nonetheless, when cells were seeded in a collagen type I biodegradable scaffold and implanted in a critical-sized bone radial defect in mice, tiMSCs presented increased bone volume density compared to the other cell types tested [41].

iMSC also promoted bone repair in an experimental model of steroid-induced femoral head osteonecrosis. The cells were implanted into the bone marrow cavity after the induction of osteonecrosis. Micro-CT and histological analyses demonstrated that iMSC had a similar capacity for bone repair and angiogenesis as BMMSC [48]. iMSC implantation in bone calvarial defects could promote de novo bone formation and increase bone volume. iPSC used to generate the iMSC were cultivated in a defined and feeder-free system suitable to use in clinical applications [42].

Another preclinical study in thoroughbred horses demonstrated that iMSC were relatively safe when injected in horses and could alleviate symptoms of musculoskeletal disorders such as osteochondritis, arthritis, and tendonitis. Some animals (25%) developed adverse effects after transplantation, including edema, flushing, and lameness. Thus, iMSC were treated with TGF-β, which can downregulate MHC-I expression by 75% and 50% at the mRNA and protein levels, respectively. Two animals were injected with iMSC treated with TGF-β and no adverse effects occurred [51].

iMSC repair bone in critically sized defects in large animal models. The regenerative potential of these cells was compared with that of bone marrow cells and autografts in vivo. iMSC obtained from human foreskin fibroblasts and autologous bone marrow cells were loaded onto calcium phosphate granules and transplanted into critical-sized defects in the proximal tibia of minipigs. Autografts were also transplanted into animals. This study demonstrated that iMSC could promote new bone formation, as evaluated by histometric analysis and tomography performed comparably to bone marrow cells in bone defects [44]. 

The therapeutic potential of iMSC for skeletal muscle regeneration was demonstrated in a mdx murine model, which is commonly used for studying muscular dystrophy in the context of Duchenne muscular dystrophy (DMD). Oxidative stress was reduced, and dystrophin expression levels were restored in skeletal muscles 6 weeks after iMSC transplantation into the injured tibialis anterior skeletal muscle of mdx mice [52]. 

**Figure 1:**
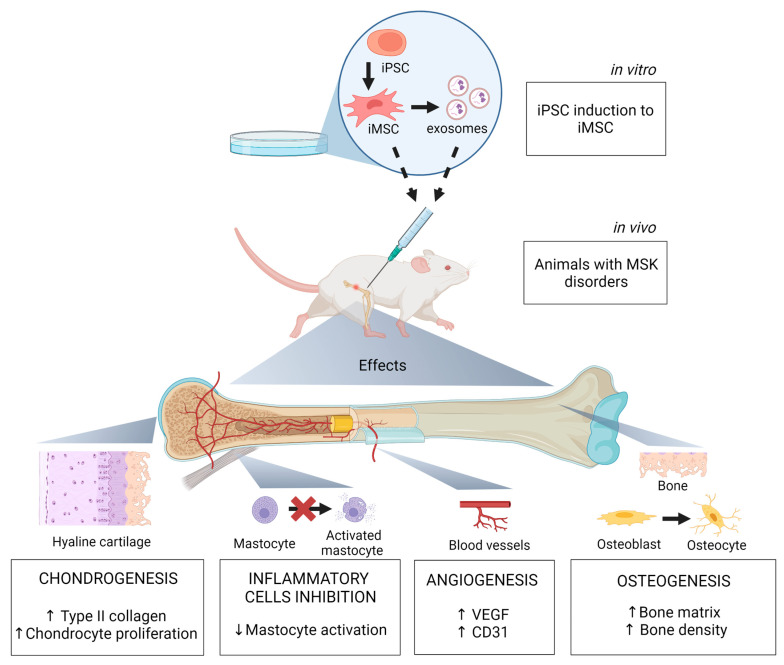
iMSC effects on experimental models of musculoskeletal diseases. iPSC obtained from healthy individuals are differentiated in vitro in iMSC. iMSC and/or their exosomes exert effects in animals with MSK disorders in vivo. These effects include: chondrogenesis through increased chondrocyte proliferation and enhanced type II collagen formation; inflammatory cells inhibition via decreasing mastocyte activation; angiogenesis via increased expression of VEGF and CD31; and osteogenesis via enhancing the bone matrix and bone volume density. iPSC, induced pluripotent stem cells; iMSC, induced pluripotent stem cells-derived mesenchymal stem cells; MSK, musculoskeletal disorders; VEGF, vascular endothelial growth factor; CD31, cluster of differentiation 31. Image created using BioRender.com.

In summary, data obtained from preclinical studies using iMSC for MSK disorders (Table 1) showed that iMSC transplantation promoted angiogenesis, osteogenesis, and chondrocyte proliferation, increased type II collagen in the extracellular matrix of articular cartilage, inhibited mast cell proliferation and M1 macrophage infiltration in vivo, and lowered oxidative damage (Figure 1). These positive effects encourage clinical studies using iMSC to evaluate their therapeutic potential for treating MSK disorders in humans.

Although no clinical trials have reported the results of iMSC transplantation in patients with MSK disorders, a patented product compatible with clinical use has been developed using iMSC. Cynata Therapeutics is an Australian stem cell and regenerative medicine company that uses a stem cell platform called Cymerus to manufacture mesenchymoangioblasts (MCA) from iPS. The MCA is used to manufacture MSC therapeutic products. The first clinical study (phase I) utilizing this type of cell was completed in 2020. Patients with acute steroid-resistant graft versus host disease (aGvHD) received two doses spaced weekly. The outcomes measured were complete, and partial responses and overall survival were based on the GvHD grade status before and after treatment. No serious adverse events were related to the administration of cells, and at day 100 after treatment, the overall response, complete response, and overall survival rates were 86.7%, 53.3%, and 86.7%, respectively [79]. A phase III, multicenter, randomized, double-blind, placebo-controlled clinical trial will be conducted in 440 patients with symptomatic tibiofemoral knee osteoarthritis. Patients will receive iMSC intra-articularly. The protocol has been published and the primary outcomes will be published within the next few years [83].

## 6. Concluding Remarks and Future Directions

The therapeutic potential of iMSC has been demonstrated in several animal studies and presents several advantages and disadvantages compared to MSC. In summary, iMSC elicit greater cell proliferation in injured tissues and present a highly proliferative and rejuvenated phenotype with less senescence than MSC. Furthermore, heterogeneity can be minimized by using a single iPSC donor from master cell banks. Nevertheless, iMSC have some disadvantages compared to MSC, such as a higher cost of implementation and an elevated risk of tumor development. Although iMSC are expected to have a higher risk of tumorigenesis because of their derivation from iPSC, none of the aforementioned in vivo studies have reported tumor formation. These cellular properties must be considered in clinical applications. Creating a standard protocol that follows good clinical practices and guidelines requires a stable cell source with low variability. Because a single healthy cell donor can generate limitless iPSC to derive MSC for allogeneic use, these cells seem to be adequate for a clinical protocol with an increased probability of consistent clinical outcomes. However, high costs and technical issues for generating iPSC must be considered because the procedure is labor-intensive and requires expensive reagents and expert technicians. However, once a master cell bank is established, costs tend to decrease. Regarding the treatment of MSK disorders, the specific characteristics of the disease must be considered. Osteoarthritis is one of the most common MSK disorders, with high prevalence and incidence in the elderly population. When autologous cell therapy is considered in older osteoarthritic patients, an MSC source, such as adipose tissue or bone marrow, should be avoided owing to aging-related microenvironments and inflamed-aging factors produced by these cells [84]. In this situation, iMSC, even from an autologous source, are a better option because they have a juvenile phenotype.

The field of regenerative medicine for osteodegenerative diseases and fractures with extreme bone loss will benefit in the near future from major advances in 3D bioprinting, an additive manufacturing technology that has the potential to produce complex engineered tissues from modular units. Relevant clinical therapies based on cells and growth factors that guide cell functions can be embedded in hydrogels to form bioinks for bioprinting bone substitutes. Indeed, a recent study reported cranial bone fracture repair with 3D-printed bone constructs fabricated from iMSC overexpressing BMP-6 in a printable bioink [85]. Eight weeks after grafting, the bone volume increased, and partial bridging between the implant and the host tissue was observed. This study paves the way for new therapeutic approaches that combine iMSC and 3D bioprinting technologies for the treatment of MSK disorders.

Moreover, for a broader translation into practice and clinical use, robust standard protocols must be implemented to ensure consistency and reproducibility. Furthermore, differentiation protocols need to ensure that all iPSC have differentiated into MSC to decrease tumorigenic potential. The results of the first clinical trial (phase I) using iMSC in GvHD showed promising outcomes [49,79]. Therefore, clinical studies using iMSC in patients with knee osteoarthritis was conducted. The experimental design and protocols were published in 2021, and the publication of the final results is scheduled for December 2024 [83]. Although clinical trials are already underway, further research is needed to address the potential risks and to ensure safety and efficacy in the treatment of musculoskeletal disorders.

## Figures and Tables

**Table 1:** Summary of preclinical studies testing the therapeutic potential of iMSC in musculoskeletal disorders.

References	iPSC Source	iMSC Differentiation Method	Animal Model	MSK Disorder	Effects/Mechanisms
[37]	Fibroblast	MSC Switch	Sprague Dawley Rats injected with 4% carrageenan	Tendinopathy	iMSC exosomes inhibit mast cells activation and their interaction with nerve fibers via the HIF-1 signaling pathway
[38]	Fibroblast	MSC Switch	Sprague Dawley Rats injected with 4% carrageenan	Tendinopathy	iMSC-EV alleviate inflammation (reduced proinflammatory cytokines) and inhibit capillary proliferation
[39]	Fibroblast	MSC Switch	Sprague Dawley Rats injected with 4% carrageenan	Tendinopathy	iMSC-lEV attenuate pain and inflammation by regulating the p38 MAPK signaling pathway
[40]	Fibroblast	MSC Switch	Sprague Dawley Rats with intervertebral caudal disc puncture	Intervertebral disc degeneration	iMSC-sEV rejuvenate nucleus pulposus cells and attenuate intervertebral disc degeneration
[41]	Fibroblast	Embryoid Body Formation	NOD/SCID mice	Nonunion radial fracture	iMSC transfected with BMP-6 induced ectopic bone formation by increasing bone volume density
[42]	Fibroblast	Embryoid Body formation	SCID mice	Craniofacial bone defect	iMSC promoted new bone formation in mice with calvarial defects
[43]	Fibroblast	Specific Differentiation	NOJ male mice with anterior cruciate ligament transection	Osteoarthritis	iMSC generated by mesodermal and neuroepithelium differentiation suppressed the degeneration of knee cartilage
[44]	Fibroblast	Pathway inhibitor and MSC switch	Goettingen mini-pigs	Critical-sized defect of proximal tibia	iMSC loaded on calcium phosphate granules promote new bone formation on the central defect area
[45,46,47]	Adult bone marrow CD 34+	Embryoid Body Formation	Athymic nude rats	Critical-sized cranial defect	iMSC seeded on CPC scaffolds increased blood vessel density and promoted de novo bone formation; effects were increased with co-seeded endothelial cells.
[48]	HEK293T	Embryoid body formation	Steroid-induced ONFH Sprague Dawley Rats	Osteonecrosis of femoral head (ONFH)	iMSC promote bone repair by forming new dense trabecular bones and increase angiogenesis by elevating VEGF and CD3 expression in the femoral head
[49]	-	MSC Switch	Collagen-induced osteoarthritis in C57B/L10 mice	Osteoarthritis	iMSC exosomes stimulate chondrocyte migration and proliferation; increased expression of collagen type II in superficial and deep zones of articular cartilage
[50]	-	MSC Switch	Bilateral ovariectomy Sprague Dawley Rats	Critical-sized bone defects in osteoporosis	iMSC exosomes associated with β-TCP scaffolds improve bone regeneration through osteogenesis and angiogenesis
[51]	Adipose derived Stem Cell	MSC Switch	Thoroughbreds horses	Fracture, arthritis, tendonitis, and osteochondritis	iMSC reduce lameness, fever, and fracture lines
[52]	-	Embryoid Body Formation	C57BL/10 mdx mice (injured tibialis anterior skeletal muscle)	Duchenne Muscular Dystrophy	iMSC diminish cellular stress related to reactive oxygen species and restor dystrophin expression in muscle cells
[53]	-	-	Nude mice	Radial bone defect	Chondrogenic pellets derived from iMSC promote high rate of bone union and transition from hypertrophic cartilage to newly formed woven bone

iPSC, induced pluripotent stem cell; iMSC, induced pluripotent stem cell-derived mesenchymal stem cell; EV, Extracellular Vesicles; sEV, small Extracellular Vesicles; lEV, large Extracellular Vesicles; MSK, musculoskeletal disorder; CPC, calcium phosphate cement; HIF-1, Hypoxia Inducible Factor-1; NOD/SCID, non-obese diabetic/severe combined immunodeficiency; -TCP, -tri-calcium phosphate; CD, cluster of differentiation; BMP, bone morphogenetic protein.

## Data Availability

No new data were created or analyzed in this study. Data sharing is not applicable to this article.
